# Synthesis and Characterization of PLA/Biochar Bio-Composites Containing Different Biochar Types and Content

**DOI:** 10.3390/polym17030263

**Published:** 2025-01-21

**Authors:** Katerina Papadopoulou, Panagiotis A. Klonos, Apostolos Kyritsis, Evangelia Tarani, Konstantinos Chrissafis, Ondrej Mašek, Konstantinos Tsachouridis, Antonios D. Anastasiou, Dimitrios N. Bikiaris

**Affiliations:** 1 Laboratory of Polymer Chemistry and Technology, Department of Chemistry, Aristotle University of Thessaloniki, 54124 Thessaloniki, Greece; katerina_1991papa@hotmail.com (K.P.); pklonos@central.ntua.gr (P.A.K.); 2 Department of Physics, National Technical University of Athens, Zografou Campus, 15780 Athens, Greece; akyrits@central.ntua.gr; 3 Laboratory of Advanced Materials and Devices, Department of Physics, Aristotle University of Thessaloniki, GR-54124 Thessaloniki, Greece; etarani@physics.auth.gr (E.T.); hrisafis@physics.auth.gr (K.C.); 4 UK Biochar Research Centre, School of GeoSciences, University of Edinburgh, Alexander Crum Brown Road, Edinburgh EH9 3FF, UK; ondrej.masek@ed.ac.uk; 5 Department of Chemical Engineering, University of Manchester, Manchester M1 9PL, UK; konstantinos.tsachouridis@manchester.ac.uk (K.T.); antonios.anastasiou@manchester.ac.uk (A.D.A.)

**Keywords:** poly(lactic acid), biochar, bio-composites, in situ polymerization, mechanical properties, segmental mobility, thermal properties, enzymatic hydrolysis

## Abstract

A series of poly(lactic acid) (PLA)/biochar (BC) bio-composites filled with low amounts (1–5 wt%) of BC were prepared and characterized. The synthesis involved the in situ ring-opening polymerization (ROP) of lactide in the presence of two different types of BC named SWP550 and SWP700, having been produced by pyrolysis of softwood pellets at two different temperatures, 550 and 700 °C, respectively. The bio-composites were characterized by complementary techniques. The successful synthesis of PLA and PLA/BC bio-composites was directly demonstrated by the formation of new bonds, most probably between PLA and BC. Indirect evidence for that was obtained by the systematic molar mass reduction in the presence of BC. BC was found by transmission electron microscopy (TEM) micrographs to be well dispersed at the nanosize level, indicating that in situ polymerization is a technique quite efficient for producing bio-composites with finely dispersed BC additive. The molecular dynamics mapping is performed here via dielectric spectroscopy, moreover, for the first time in these PLA/BC systems. The strong PLA/BC interactions (due to the grafting) led to a systematic deceleration of segmental mobility (elevation of the *T*_g_) in the bio-composites despite the opposite effect expected by the decrease in molar mass with the BC content increasing. In addition, the same interactions and chain-length reduction are responsible for the slight suppression of the PLA’s crystallizability. The effects are slightly stronger for SWP700 as compared to SWP550. The crystal structure is rather similar between the unfilled matrix and the bio-composites, whereas, based on the overall data, the semicrystalline morphology is expected to be tighter in the bio-composites. The thermal stability and decomposition kinetics were also thoroughly studied. All materials exhibit good resistance to thermal degradation. Additionally, the mechanical properties of bio-composites were evaluated by tensile testing and found slightly enhanced at low biochar contents and decreasing thereafter due to the low molecular weight of bio-composites with the larger BC contents.

## 1. Introduction

Global plastics production was estimated at 150 million metric tons in 2023. This is expected to rise in the following decades, reaching approximately 590 million metric tons by 2050 [1]. Therefore, the establishment of a bio-economy, as well as a circular economy, is related, among others, to the development of biobased and biodegradable plastics, i.e., alternative plastics made of renewable resources and eco-friendly in order to contribute towards the progressive independence from fossil-derived sources.

The urge to replace petroleum-based polymers has expectedly increased the interest in eco-friendly and sustainable materials. Poly(lactic acid) (PLA) is a very highly promising material for a wide range of applications, such as in medicine, packaging, agriculture, electronics, automotive, etc. [2,3,4], due to its biobased origin. PLA is an aliphatic polyester, which is produced through the fermentation of sugars obtained from sugarcane, corn starch, or potato starch and then converted to lactic acid [2,3]. PLA is typically synthesized by ring-opening polymerization (ROP) of L-lactide utilizing organometallic catalysts and organic solvents [2,5]. Over recent years, PLA has been one of the most produced and used biobased plastics in the world [6]. Moreover, depending on its structure, namely molar mass, and stereoisomerism (L-/D-lactide ratio), PLA is semicrystalline [7]. This presents both advantages and disadvantages, as crystallinity can be a severe parameter on many aspects regarding the polymer performance (mechanical strength, permeation of small molecules [8], thermal conductivity [9,10], compostability [11], etc.). However, PLA presents some drawbacks, such as low melt strength, fragility, and, thus, limited mechanical properties, moisture, and gas sensitivity [12,13]. These drawbacks can be overcome by using several fillers and nano additives [14].

The addition of organic/inorganic fillers provides a potential solution to the limitations of PLA, with notable enhancements in mechanical, thermal, and many other physical properties. In recent years, biochar (being introduced below) has been used as a biobased filler in order to improve the aforementioned properties of polymers. In particular, the thermomechanical properties of PLA-biochar composites were investigated with two different process methods (melt mixing and solvent casting) by Arrigo et al. [15]. In that study, the processing method indicated a remarkable influence on the thermal behavior of the bio-composites, and the addition of biochar decreased their thermal stability. Furthermore, Hernandez-Charpak et al. studied the effect of two different biochar feedstocks on the morphological, thermal, and mechanical properties of three polymeric matrixes, one of them being PLA [16]. Therein, it was investigated that different types of biochar affected mechanical and thermal properties in various ways. The effect was found to be different depending on the polymeric matrix and the biochar feedstock.

The bio-derived carbon, which is well known as biochar (BC), is a promising replacement for conventional carbonaceous non-renewable fossil-based fillers, such as carbon tubes, carbon black, carbon nanotubes, and graphene [17,18,19]. BC is a byproduct of biomass pyrolysis at high temperatures (300–800 °C). It is an abundant material prepared from many types of biomass plants. It is an actual cost-effective, non-toxic, eco-friendly, and sustainable material [20]. BC is characterized by a large surface area and stable structure with, obviously, high carbon contents [21]. The structure as well as the properties of BC are determined by numerous factors, including the type of feedstock, conditions of pyrolysis, and particle size [22]. For instance, with the increase in pyrolysis temperature from 550 to 750 °C, the carbon percentage of biochar is increased, the number/concentration of carboxylic groups is reduced, its hygroscopicity can be altered, and its total surface area can be increased [23,24]. In general, mechanical properties, thermal stability, electrical conductivity, and hydrophilicity or hydrophobicity of the materials could be affected by the type of biomass and the pyrolysis conditions [25,26,27,28]. The effect of BC on the mechanical, rheological, and morphological properties of PLA and poly(butylene adipate-co-terephthalate) (PBAT) blends has been carried out by Giulia Infurna et al. [29]. Also in our previous studies, we found that the addition of low amounts of biochar (1 and 2.5 wt%) in poly(butylene succinate) improved significantly the tensile and impact strengths [30] and enhanced its resistance against UV irradiation [31]. Moreover, we revealed that the BC effects of the polymer can be both direct and indirect, for example, via altering crystallinity.

The aim of the present study is to study the incorporation of different types and amounts of biochar in the PLA polymeric matrix. For this reason, we synthesized PLA/BC bio-composites with biochar’s feedstock softwood pellet (SWP), having been produced at two different pyrolysis temperatures (550 °C and 700 °C), employing the in situ ring-opening polymerization technique. Biochar was incorporated for the first time by in situ ROP procedure into PLA at different weight contents (1, 2.5, and 5 wt%), aiming at evaluating the effect of different biochar types and loadings on the chemical structure, mechanical properties, molecular mobility, enzymatic hydrolysis, and thermal stability of PLA.

## 2. Materials and Methods

### 2.1. Materials

L-lactide (LA) monomer Purlact B3 (purity 99% *w*/*w*, stereochemical purity in L-isomer 95%) was purchased from Corbion N.V. (Gorinchem, The Netherlands). Tin(II) 2-ethylhexanoate (Sn(Oct)_2_) (purity > 92.5%) was purchased from Merck KGaA (Darmstadt, Germany). The biochar (BC) used in this work was produced by intermediate pyrolysis of pelleted softwood pellets at 550 and 700 °C in a pilot-scale rotary kiln at the UK Biochar Research Centre, referred to as SWP550 and SWP700, respectively [32]. Prior to its use, the BC was dried overnight in the oven at 80 °C under vacuum. All other reagents were of analytical grade.

### 2.2. Synthesis of PLA and Its Bio-Composites

PLA and its bio-composites with biochar were synthesized by in situ ROP of lactide in the presence of Sn(Oct)_2_. The catalyst was added as a 400 ppm solution in toluene. Dodecanol (0.05 g/mL acetone) was added as a co-initiator. The polymerization was carried out at 160 °C for 1.5 h with a 200 rpm screw speed and with a flow of dry N_2_ in order to minimize the presence of air in the reaction chamber (250 mL round-bottom flask). After 1.5 h, the temperature increased to 180 °C, and the polymerization was continued for an additional 15 min under vacuum to remove untreated lactide monomer. The polymerization was terminated by rapid cooling of the flask to room temperature. Similarly, PLA/BC bio-composites were prepared to contain 1, 2.5, and 5 wt% biochar using the two different types, SWP550 and SWP700, which were added at the beginning of the reaction with lactide in the batch reactor. In total, 7 samples were synthesized.

### 2.3. Intrinsic Viscosity

Intrinsic viscosity [*η*] measurements were carried out using an Ubbelohde viscometer capillary 0c at 25 °C in chloroform. The solutions were filtered through a disposable membrane filter (Teflon). The average value was calculated after three different measurements. The intrinsic viscosity value of the polymer was calculated by the Solomon–Ciuta Equation (1):

(1) η=2tt0−lntt0−112C

where *c* is the solution concentration, *t* is the flow time of the solution and *t*_0_ is the flow time of pure solvent.

### 2.4. Gel Permeation Chromatography-Size Exclusion Chromatography (GPC/SEC)

The molecular weight of the materials was determined using GPC/SEC analysis. The analysis was conducted on an Agilent 1260 Infinity II LC system (Agilent Technologies, Santa Clara, CA, USA), which included an isocratic G7110B pump, an automatic vial sampler G7129A, a refractive index detector (RID) G7162A, a PLgel 5 μM (50 × 7.5 mm) guard column, and two PLgel 5 μM (300 × 7.5 mm) MIXED-C columns. Calibration was performed using poly(methyl methacrylate) (PMMA) standards with molecular weights ranging from 0.535 to 1591 kg/mol. Samples were prepared by dissolving them in CHCl_3_ at a concentration of 3 mg/mL and filtering the solution through a 0.45 µm PTFE microfilter to eliminate any solid residues. Each sample was injected in a 20 μL volume, with a total elution time of 30 min. Both the column and RID temperatures were maintained at 40 °C throughout the analysis.

### 2.5. Nuclear Magnetic Resonance (NMR)

NMR spectra of aliphatic polyesters were recorded in deuterated chloroform. An Agilent 500 spectrometer (Agilent Technologies, Santa Clara, CA, USA) was used for the structural study of the bio-composites at room temperature. Spectra were calibrated using the residual solvent peaks.

### 2.6. Fourier-Transformed Infra-Red Spectroscopy (FTIR)

The FTIR spectra for the two types of biochar and the produced materials were obtained by FTIR-2000 (Perkin Elmer, Waltham, MA, USA). All spectra were collected in the wavenumber range from 450 to 4000 cm^−1^ using a resolution of 4 cm^−1^ and 32 co-added scans. The presented spectra were further baseline corrected, normalized, and converted into an absorbance mode.

### 2.7. Transmission Electron Microscopy (TEM)

TEM analysis was conducted using an FEI Tecnai G2 20 microscope (FEI, Hillsboro, OR, USA) operating at an accelerating voltage of 200 kV. For sample preparation, thin films of neat PLA and its bio-composites were sectioned using an ultramicrotome equipped with a DiATOME 45° diamond knife (DiATOME Ltd., Nidau, Switzerland) to achieve a thickness of 80 nm. The resulting sections, which floated on the water surface of the knife, were subsequently transferred onto carbon-coated grids and allowed to air dry overnight.

### 2.8. X-Ray Diffraction (XRD)

The XRD measurements were performed using a MiniFlex II XRD system (Rigaku Co., Tokyo, Japan) in the angle 2θ range from 5° to 45° with a scanning speed of 1°/min with CuKα radiation (λ = 0.154 nm). All samples were in the form of films.

### 2.9. Differential Scanning Calorimetry (DSC)

The thermal transitions of PLA (glass transition, crystallization, melting) were studied using a TA Q200 DSC instrument (TA Instruments, New Castle, DE, USA), priorly calibrated with Indium for temperature and enthalpy and with sapphires for heat capacity. The measurements were performed in a gas nitrogen atmosphere of high purity (99.9995%) and within the temperature range from −20 to 190 °C on sample pieces of ~7–9 mg in mass closed in TA Tzero aluminum pans. In order to erase the thermal history of the sample, a first heating scan up to 190 °C was performed. Subsequently, the sample was cooled from 190 °C (melt state) (scan 1) down to −20 °C at the fastest achievable rate (~100 K/min), stayed there for 5 min, and heated up to 190 °C at 10 K/min; then, (scan 2) the sample was cooled from 190 °C (a, scan 1) down to −25 °C at 20 K/min, stayed there for 2 min, and, finally, heated to 190 °C at 10 K/min.

### 2.10. Dielectric Relaxation Spectroscopy (DRS)

Segmental mobility was studied employing the advanced technique of DRS [33]. The measurements were performed using a Novocontrol DRS setup (Novocontrol GmbH, Montabaur, Germany) in a nitrogen atmosphere on samples in the form of sandwich-like capacitors of 15 mm in diameter and ~100 μM in thickness (silica spacers employed). The samples were melted at ~180–190 °C between the finely polished brash electrodes (disks) and were subsequently cooled rapidly in order to produce PLA in the amorphous state. For this study, we recorded and evaluated the imaginary part of dielectric permittivity, *ε*″, related to the dielectric losses, as a function of frequency in the range from 10^−1^ to 10^6^ Hz and in the temperature range between 10 and 120 °C, upon heating at steps of 5 or 10 K.

### 2.11. Mechanical Properties

Tensile tests of PLA and its composites were performed using a Shimadzu EZ Test Tensile Tester (Kyoto, Japan), Model EZ-LX, with a 2 kN load cell, in accordance with ASTM D882 using a crosshead speed of 5 mm/min. Dumbbell-shaped tensile test specimens (central portions 5 × 0.5 mm thick, 22 mm gauge length) were prepared by compression molding in a thermopress at 170 °C, cooled rapidly, and cut in a Wallace cutting press. At least five measurements were conducted for each sample, and the results were averaged to obtain the mean values of Young’s modulus, tensile strength at yield and breakpoint, and elongation at break.

### 2.12. Color Measurement

A Datacolor Spectraflash SF600 plus CT UV reflectance colorimeter (Datacolor, Marl, Germany) using the D65 illuminant was used for color measurements, including a 10° standard observer with the UV component excluded and the specular component. The coloration of the bio-composites was analyzed using the CIEL*a*b* color system. In this system, the L* axis represents luminosity or lightness, with values ranging from 0 (black) to 100 (white). The a* coordinate indicates redness when positive and greenness when negative, while the b* coordinate signifies yellowness when positive and blueness when negative. Additionally, C* denotes chroma, and H* refers to the hue angle. To evaluate the color concentration in the bio-composites, the K/S ratio was calculated [34].

### 2.13. Enzymatic Hydrolysis

Film samples, each measuring 1.5 cm × 1.5 cm with an approximate thickness of 1 mm, were prepared using an Otto Weber Type PW30 hydraulic press (Wuppertal, Germany). These films were placed in test tubes containing 10 mL of phosphate buffer solution (0.2 M, pH 7.2 ± 0.1) supplemented with 0.09 mg/mL of *Rhizopus delemar* lipase and 0.01 mg/mL of *Pseudomonas cepacia* lipase. The test tubes were incubated in an oven at 50 ± 1 °C for one month, with the buffer/enzyme solution being refreshed every three days. At specified intervals (5, 10, 15, 20, 25, and 30 days), the films were retrieved from the solution, thoroughly washed with distilled water, and dried under vacuum at room temperature until a constant weight was achieved. Each measurement was conducted in triplicate. The rate and extent of enzymatic hydrolysis were determined based on the mass loss of the samples according to the following equation:

(2) $$Mass\;loss\;\%=\frac{W_{0}-W_{i}}{W_{0}}$$

Alterations of the morphology of the films after enzymatic hydrolysis were examined using SEM JEOL (JMS 760F).

### 2.14. Water Contact Angle

The water contact angle was measured with an Ossila contact angle goniometer L2004A1 at room temperature (25 °C). The contact angle was measured by gently placing a water droplet (5 µL) on the surface of the films of the samples prepared by in situ polymerization. At least three measurements were performed for each sample, and the mean value was reported.

### 2.15. Scanning Electron Microscopy (SEM)

The particle size of used BC and the surface morphology of enzymatically hydrolyzed materials were studied with a JEOL (Tokyo, Japan) JSM 7610F field emission scanning electron microscope operating at 5 kV. Pictures of thin films were also captured using a Jenoptik (Jena, Germany) ProgRes GRYPHAX Altair camera attached to a ZEISS (Oberkochen, Germany) SteREO Discovery V20 microscope.

### 2.16. Thermogravimetric Analysis (TGA)

Thermogravimetric analysis (TGA) of PLA/BC bio-composites was performed by a SETARAM SETSYS TG-DTA 16/18 instrument. The samples (10 ± 0.5 mg) were placed in alumina crucibles, while an empty alumina crucible was used as a reference. For the kinetic analysis study [35]. PLA composites were from 25 °C to 600 °C, and the N_2_ at the rate of 50 mL/min flow for heating rates of 5, 10, 15, and 20 °C/min. Thermal degradation kinetic analysis of the PLA composites was achieved with the use of NETZSCH Kinetics Neo software (NETZSCH, Selb, Germany) [36]. Among the model-fitting kinetic approach algorithms is the deterministic one that seeks the best kinetic model by changing minimal parameters quantitatively describing the kinetics of the complete degradation reaction.

## 3. Results

### 3.1. Synthesis and Morphological Characterization of Biochar

**Figure 1:**
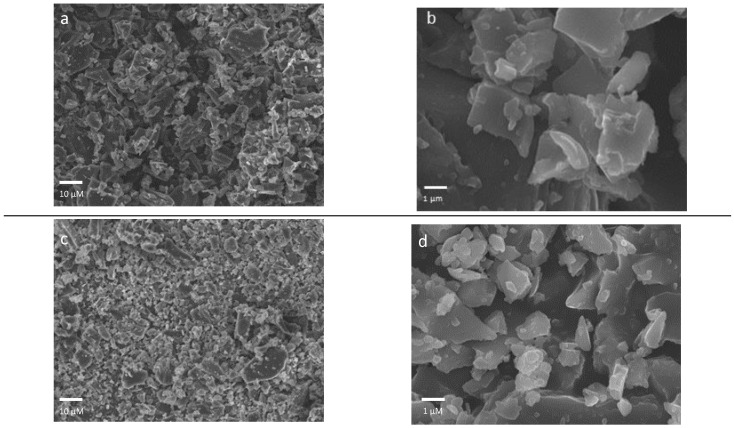
SEM micrographs of prepared biochar after pyrolysis at 550 and 700 °C and grinding in low (left) and high (right) magnification. Figures (**a**,**b**) depict biochar pyrolyzed at 550 °C, while figures (**c**,**d**) depict biochar pyrolyzed at 700 °C.

Biochar from softwood pellets (SWP) was produced by intermediate pyrolysis at 550 and 700 °C in a pilot-scale rotary kiln [32]. After grinding, irregular particle sizes and shapes of biochar were formed. The SEM micrographs of Figure 1 show both large particles with sizes greater than 10 µm (10–15 µm) and much smaller particles with sizes at the nanoscale level (250–500 nm). These particles have been used as fillers for PLA/biochar bio-composites production.

### 3.2. Synthesis and Characterization of In Situ Prepared PLA/BC Bio-Composites

Neat PLA and its bio-composites were prepared by in situ ring-opening polymerization, yielding samples with high molecular weight. The number average molecular weight, M_n_, values of neat PLA are evaluated by GPC as 109 kg/mol (Table 1), which is high for aliphatic polyesters. However, as biochar content increases in the polymeric matrix, the M_n_ is reduced. Usually, when additives have some reactive groups, they can interact with the polymer matrix, affecting its molecular weight [16,18,37,38,39]. This can be attributed to the hydroxyl groups of biochar [19], which can act as co-initiators in ROP. The creation of new initiation sites can lead to lower molecular weight values. We recall that the thermochemical and time period parameters of ROP were kept fixed. Thus, compared to neat PLA, the drop in M_n_ is related to the simultaneous initiation of ROP at more sites in the presence of BC. Thus, M_n_ of PLA/SWP550 with 1, 2.5, and 5 wt% are about 40, 30, and 22 kg/mol, respectively, and M_n_ PLA/SWP700 with 1, 2.5, and 5 wt% are about 40, 31, and 20 kg/mol, respectively. This significant drop in M_n_ can be used as indirect, although strong, proof of the successful PLA grafting onto the BC entities. Interestingly, the smaller polydispersity indices (PDI) exhibited by the bio-composites (1.5–1.8), as compared to the unfilled PLA (2.2), suggest a moderate improvement in the bio-composites homogeneity. The intrinsic viscosity [η] was also calculated in order to confirm the M_n_ values alternations qualitatively. It can be seen that with the addition of biochar, the intrinsic viscosity values are decreased monotonically (Table 1), which is in good agreement with the M_n_ reduction trend calculated by GPC.

**Scheme 1:**
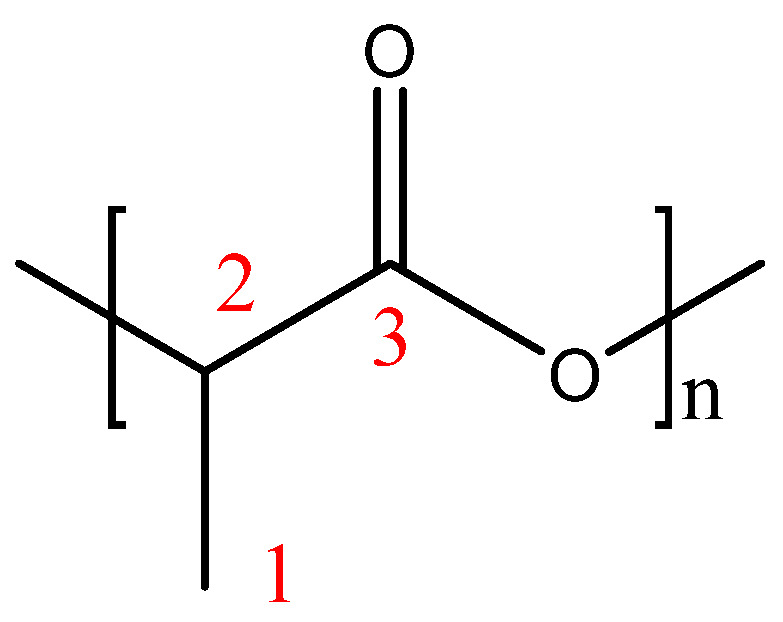
Numbered structure of the prepared PLA and PLA/BC bio-composites.

**Figure 2:**
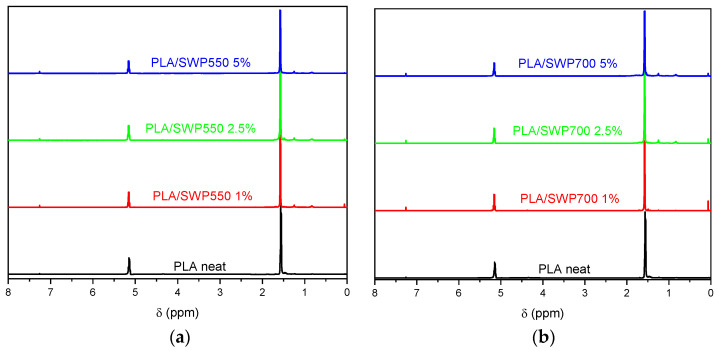

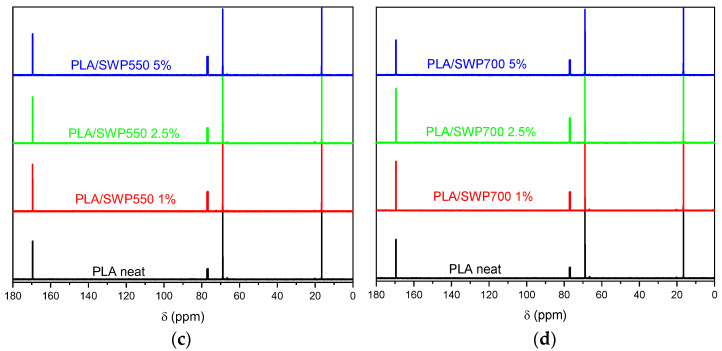
(**a**) ^1^H NMR of PLA/SWP550, (**b**) ^1^H NMR of PLA/SWP700, (**c**) ^13^C NMR of PLA/SWP550, and (**d**) ^13^C NMR of PLA/SWP700.

The NMR spectra were obtained in order to evaluate the successful synthesis of PLA and PLA/BC bio-composites. The chemical structure of the produced aliphatic polyesters was assessed by proton (^1^H) and carbon (^13^C) nuclear magnetic resonance spectroscopy. Scheme 1 presents the numbered structures of the studied polymers. The part of the structure corresponding to carbons 1–3 appears basically at the same shifts for all samples, and the assigned chemical shift in ^1^H and ^13^C spectra remains at approximately the same positions (Figure 2). The ^1^H-NMR resonance signal of neat PLA appears at 1.58 ppm (d, 3H, J = 7.1 Hz), is associated with the methylene protons (CH_3_-), and at 5.16 ppm (q, 1H, J = 7.1 Hz) to methine protons (-CH-), Figure 2a,b. Figure 2c,d depicts the ^13^C NMR spectra of the synthesized polymers. As mentioned above, the peaks of all samples corresponding to carbons 1–3 remain the same. At 16.1 ppm, the methyl carbons (1) are recorded, and at 68.9 ppm, the methine carbons (2). The carbons that are adjacent to the oxygen atom of the ester bond, carbonyl carbons, (3) give a peak at 169.6 ppm. The structural integrity of PLA is not compromised by the incorporation of biochar. Thus, the NMR spectra demonstrated successful synthesis of the PLA and PLA/BC composites. The results are in accordance with the literature [19,40]. The residual monomer in neat PLA and composites was also calculated by NMR, and in all cases is lower than 1 wt%. This is very important since the amount of residual lactide can affect the characteristics of the final polymer [41].

### 3.3. Structural Characterization of Biochar and PLA/BC Bio-Composites

**Figure 3:**
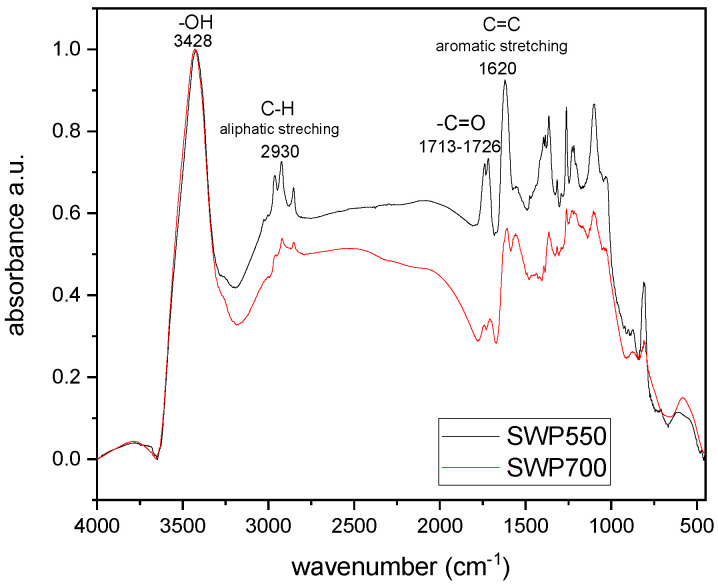
FT-IR spectra of biochar pyrolyzed at 550 °C and 700 °C.

From the FT-IR analysis of two different types of biochar (SWP550 and SWP700), their chemical structure can be seen in Figure 3. Examining the bands in the regions of interest, a characteristic peak at 1620 cm^−1^ attributed to aromatic C=C stretching and two bands at around 1700 and 2900 cm^−1^ attributed to carbonyl groups and aliphatic C-H stretching vibrations, respectively, can be seen. Furthermore, a broad band at around 3400 cm^−1^, which arises from the hydroxyl groups, indicates dehydration of cellulosic and ligneous components of softwood pellets [29,38,42,43].

**Figure 4:**
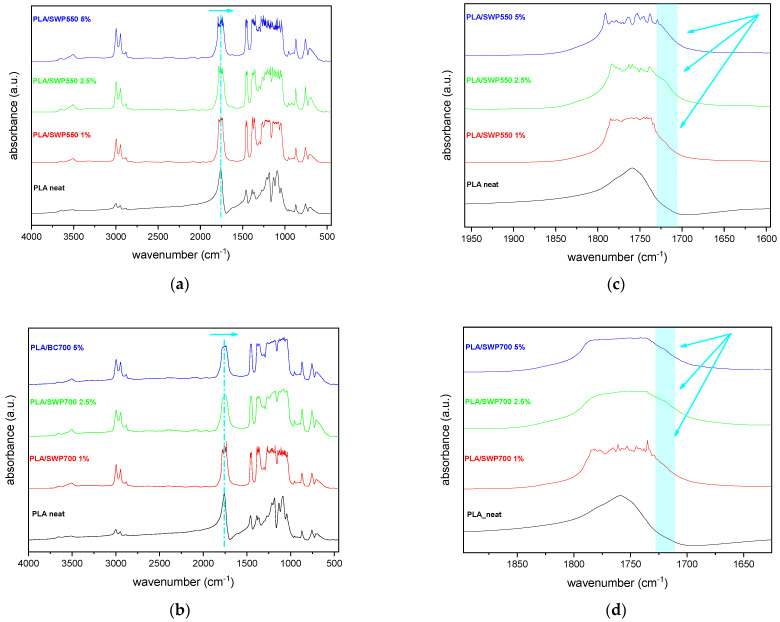
FT-IR spectra of neat PLA and its bio-composites with biochar pyrolyzed at (**a**) 550 °C and (**b**) 700 °C. Overlay of corresponding carbonyl peaks (**c**) for PLA/SWP550 bio-composites and (**d**) PLA/SWP700 bio-composites. The blue arrows mark the minor shift of bio-composite spectra toward lower wavenumbers.

Changes in the chemical structure upon the synthesis of PLA composites were evaluated through FT-IR spectroscopy. Figure 4a shows the FT-IR spectra of neat PLA and its bio-composites with SWP550 biochar, while Figure 4b displays the FT-IR spectra of PLA and its bio-composites with SWP700 biochar. In particular, the region for PLA and its bio-composites at around 1750 cm^−1^ of stretching vibration of ester groups provides information with respect to the relationship between PLA and biochar. Please note that the ester site (-C=O) is the most polar one for PLA; thus, it is the number one candidate for involvement in interactions. In the case of neat PLA spectrum, this peak at 1759 cm^−1^ is sharp, whereas, in the case of bio-composites, both SWP550 and SWP700 biochar are recording a broadening of the peak at smaller wavenumber sides. In particular, changes in the peak position and shape are recorded with the appearance of a split in the band. This is indicative of interfacial interactions between the biochar and PLA, probably due to the formation of new bonds between the -OH groups of biochar and the ester groups of PLA. Such broadening has been observed before in various polymer nanocomposites (including PLA) and has been interpreted in terms of the formation of more bound ester groups (polar sites) of the polymer due to their engagement by many polar groups of the nanofiller (e.g., -OH) [44,45]. By this result, we gain a first indication of the direct interaction-attachment of the PLA chains over the BCs, which is actually the idea behind the in situ synthesis. A small band at 3512 cm^−1^ is assigned to hydroxyl groups. This band, within all bio-composites, is increased when increasing the biochar content. It should be mentioned that the increase in the intensity of these peaks of the bio-composites may be assigned to the interactions between the fillers and the polymeric matrix. Additionally, this can be attributed to the reduced molecular weight of the bio-composites, since the smaller the molecular weight, the greater the carbonyl and hydroxyl end groups.

### 3.4. Biochar Dispersion Studied by TEM

**Figure 5:**
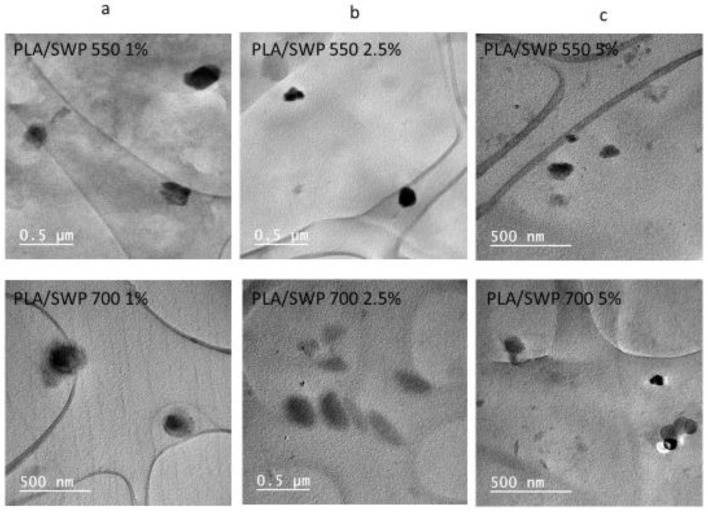
TEM micrographs of PLA/BC bio-composites with (**a**) 1 wt% biochar, (**b**) 2.5 wt% biochar, and (**c**) 5 wt% biochar.

From TEM micrographs taken of all of the composites (Figure 5), it seems that particles of BC are well dispersed throughout the PLA matrix; furthermore, in a nanosized form. Figure 5 shows dark, almost spherical particles with small sizes (100–200 nm) for the composites containing 1, 2.5, and 5 wt% of biochar with a 550 °C pyrolysis temperature. Especially for the sample of PLA/SWP550 with 5 wt% BC, there are several nanoparticulate entities of ~20–30 nm in size. Furthermore, the composites containing 1 and 2.5 wt% of biochar with a 700 °C pyrolysis temperature have irregular shapes with sizes between 200 and 300 nm. In the case of the PLA/SWP700 5 wt% composite, the appearance of the nanoparticles is the same, with sizes of 100–200 nm. It seems that some aggregates were formed with the incorporation of BC content, though remaining in the range of nanoparticles. However, from these micrographs, it is clear that even though we started from BC particles with sizes greater than 10 µm, these broke down during the in situ polymerization at the nanosized level. In this regard, the achievement of a fine dispersion of BC nanoparticles is an additional advantage of the polymerization procedure. Similar results have been found also in our previous study with poly(butylene succinate)/biochar bio-composites [30]. This reduction could be attributed to the softness of BC particles, which during in situ polymerization are broken down into smaller particles. Thus, in all cases, BC is reduced to a nanosize level.

### 3.5. XRD

**Figure 6:**
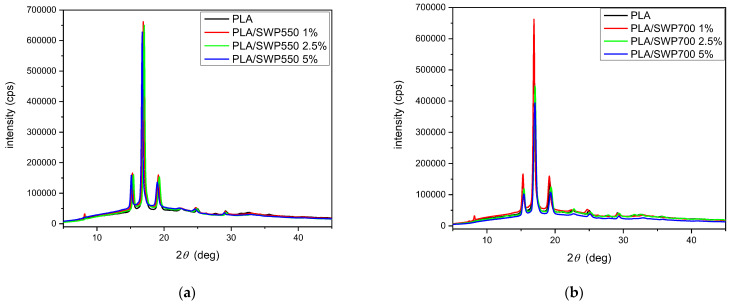
XRD patterns of neat PLA and its bio-composites at different biochar loadings: (**a**) with biochar at 550 °C, (**b**) with biochar at 700 °C.

XRD patterns of PLA and its bio-composite films upon annealing at 100 °C for 1 h were recorded in order to evaluate the impact of biochar on the crystal organization of PLA chains (Figure 6). All initial taken samples after ROP synthesis were amorphous. The diffraction peaks at 2θ = 15.2°, 16.9°, and 19.2° have been recorded. Examining the position of these peaks, it can be seen that all PLA/BCs are in similar 2θ positions, as in the case of neat PLA, which is an indication that the addition of BC does not severely affect the crystalline structure of PLA. The bio-composites show sharp peaks at the same 2θ values.

### 3.6. Thermal Transitions (DSC)

**Figure 7:**
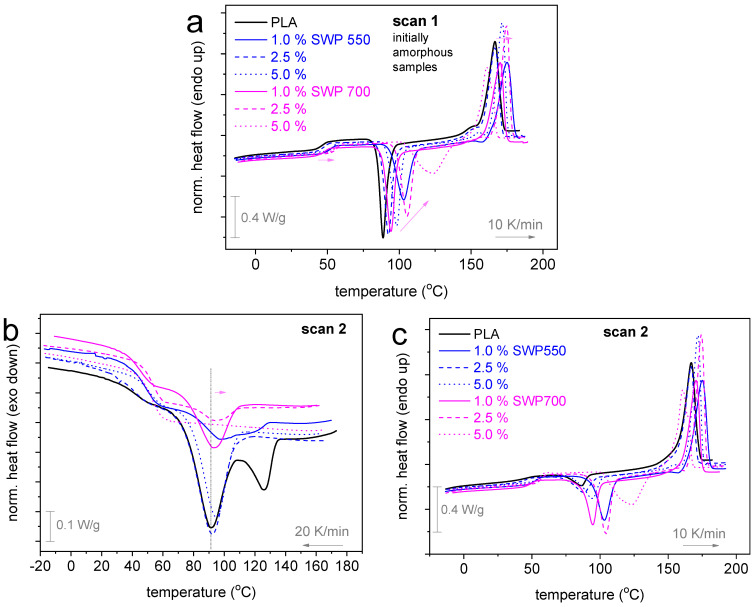
Comparative DSC traces during (**a**) the heating of scan 1 and (**b**,**c**) the cooling and heating of scan 2. The heat flow is shown upon normalization to the sample mass. The added arrows mark effects imposed by the filler addition.

In Figure 7, we present the overall DSC data for both scans 1 and 2 in the form of comparative thermograms. During the fast cooling of initially amorphous samples (scan 1, not shown), no crystallization exothermal peak was recorded. Thus, the polymers are considered amorphous. During the subsequent heating (Figure 7), all samples exhibit sharp glass transition steps (35–55 °C), cold crystallization exothermal peaks (75–140 °C), as a result of the absence of melt crystallization during cooling, and melting endotherms (150–180 °C).

Due to the amorphous character of the samples, at temperatures around the glass transition event, any alternations in the composites should be due to either the direct filler effects or/and the M_n_ alternation. The characteristic glass transition temperature, T_g_, is estimated as the T at the half heat capacity change (Δc_p_). The T_g_ values can be seen in Table 2. Compared to neat PLA, the T_g_ is increased in the composites by 1–10 K. The increase suggests the hindering of the segmental mobility of PLA. Such hindering is expected in polymer composites, especially in the implementation of strong attractive interfacial interactions [46]. The result supplements the previous discussion on FTIR (alteration in the ester group vibration). On the other hand, an opposite effect on T_g_ would be expected by the lowering of M_n_. This effect may exist here; however, it seems to be compensated by the more dominant role of interactions (grafted polymer chains).

Regarding crystallization, in scan 1, there is an elevation of the cold crystallization temperatures (T_cc_, Table 2) in PLA/BC, always comparing with neat PLA. During the melt crystallization of scan 2, the melt crystallization temperature, T_c_, decreases. Also, the estimated crystalline fraction of neat PLA is 55% for cold crystallization and 35% for melt crystallization. Both values are suppressed in the presence of BC of both types. Taking together all these effects on crystallization, we conclude that there is a decrease in crystallizability in the composites, in both the terms of nucleation and crystal formation. Such effects are compatible with previous recordings on nanocomposites with strong polymer-filler interactions [46,47]. In addition, the suppression in crystallizability is compatible as well with the reduced M_n_. The effects on the melting temperature, T_m_, (157–175 °C) are mainly minor, whereas there seems to be a tendency of T_m_ to drop for the higher BC loadings. Moreover, the endothermal event becomes complex (double peak). Comparing the two types of BC, in general, there is no severe difference in the imposed effects on the studied thermal events.

Regarding future applications and the PLA/BC performance, one would expect a significant role of BC via the alternations in the crystallizability of PLA. As discussed, the suppressed CF here is a result of the existence of strong interactions combined with the suppressed M_n_ [30,48]. It would be worth it to compare in the future the impact of BC on the crystallinity and corresponding macroscopic performance [49] of PLA or other polyesters in composites prepared by alternative mixing routes in order to bypass the change in the polymers’ M_n_ recorded herein.

### 3.7. Molecular Mobility (DRS)

**Figure 8:**
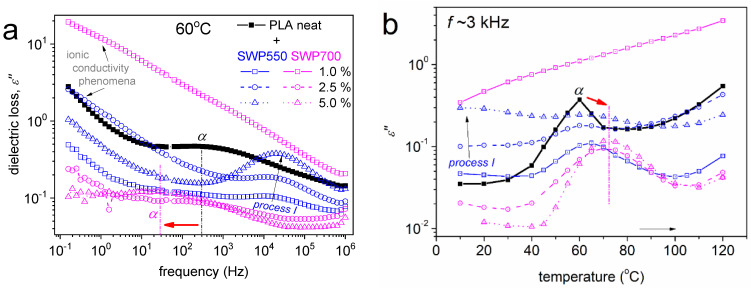
Comparative DRS results of ε″ in the form of ((**a**), raw data) isothermal curves at 60 °C and ((**b**), replotting) isochronal curves at f~3 kHz. Indicated are the main relaxation processes recorded. The added arrows mark the effects arising from the filler addition.

The advanced technique of DRS was employed to study the dynamics of glass transition, or else the so-called α dipolar relaxation, which is the dielectric analog of the calorimetric glass transition [33]. This process is recorded as a peak of the dielectric loss [ε″(f) and ε″(Τ)] at T ≥ T_g_, as shown in the selected isothermal recordings of Figure 8a and the isochronal representations of Figure 8b. Please note that in PLA/SWP550 an additional, faster process could be also resolved (named process I), resembling an additional local-like mobility. At the lower frequencies and higher temperatures, a significant signal uprise is recorded. This is due to the involvement of various ionic conductivity effects (ion transport, electrode polarization, interfacial charges, etc.). Interestingly, there is a special response of PLA/SWP700 1.0%, indicating high ionic conductivity even at T < T_g_. The result was checked and found repeatable, precluding the evaluation of molecular dynamics (strong conductivity domination over the dipolar response).

**Figure 9:**
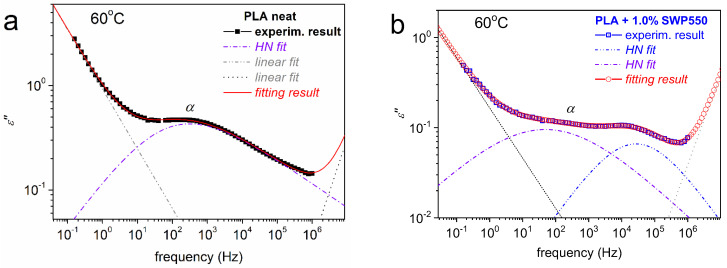
Examples of fitting of the ε″(f) spectra for the described samples [(**a**) neat PLA and (**b**) PLA + 1.0% SWP550] and temperatures, are given details in the text.

Employing well-known methods involving fitting suitable model functions (here, the asymmetric Havriliak–Negami and the symmetric Cole–Cole models) [33], we performed an analysis of the complex ε″(f) spectra. Examples of the analysis-fitting process are shown in Figure 9.

**Figure 10:**
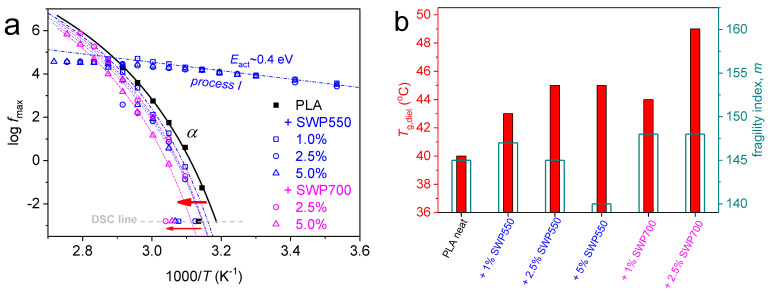
(**a**) Dielectric relaxation map in terms of time scale for all samples, described on the plot. The straight and curved lines connecting the experimental points are, respectively, fittings of the Arrhenius and Vogel–Tammann–Fulcher–Hesse equations. (**b**) The estimated dielectric T_g_ (left axis) and fragility index (right axis), from α relaxation, for the various samples.

By the performed critical analysis, we were able to construct the segmental dynamics map in terms of the reciprocal temperature (1/T) dependence of the α relaxation maximum f (f_max_). The map is presented in Figure 10a. Therein, the α relaxation exhibits, in all cases, the so-called Vogel–Tammann–Fulcher–Hesse behavior [i.e., a curved f_max_(1/T) trend], which is characteristic of segmental (cooperative) dynamics. Regarding the exceptional case of PLA/SWP550, the additional process I exhibit a linear time scale (Arrhenius-type) in Figure 10a, indicating a non-cooperative character. The estimated activation energy for process I is ~0.4 eV. Surprisingly, process I exhibits modes both ‘faster’ (at T < T_g_) and ‘slower’ (T > T_g_) than those of the α relaxation. This suggests not purely local dynamics of the polymer. Comparing with previous findings in polymers adsorbed on flat solid surfaces [50] and polyesters adsorbed on the solid surfaces of nanoparticles [51], wherein a similar dynamic view has been demonstrated, we may propose that process I arises from modified dynamics PLA chains being quite close to or adsorbed on the BC surfaces.

Coming back to segmental dynamics, all composites exhibit decelerated α dynamics, namely, migrating toward higher temperatures/lower frequencies. From these data, we evaluated the ‘dielectric glass transition temperatures, T_g,diel_’ to vary between 40 and 49 °C (Figure 10b). In all cases, there is a systematic increase in T_g,diel_ (i.e., dynamical deceleration) in the bio-composites. The effect provides additional support to the above-mentioned concept of strong chain grafting.

The time-scale data of α relaxation enable also the evaluation of the dynamical fragility index [52]; m equals 145 for neat PLA; it slightly increases in the composites with 1 and 2.5% BC, whereas it drops for the 5% SWP550 (Figure 10b). The increase/decrease in m could be an indication of increasing/decreasing of the polymer chains degree of cooperativity or else narrowing/widening of the corresponding cooperativity lengths. Especially the drop of m here is compatible with the significant reduction of the chain lengths (M_n_) in the bio-composites. More parameters are implemented within the cooperativity, such as any alternations in the free polymer volume and/or in the chain-chain associations (entanglements).

### 3.8. Mechanical Properties of PLA/BC Bio-Composites

Table 3 shows the results for the tensile properties of neat PLA and its bio-composites with SWP550 and SWP700 and with different biochar content, 1, 2.5, and 5 wt%. The tensile strength of neat PLA is about 30 MPa; PLA/SWP550 and PLA/SWP700 with 1 wt% BC are about 32 and 31 MPa, respectively. The tensile strength of PLA/SWP550 with 2.5 wt% BC is about 15 MPa, and PLA/SWP700 with 2.5 wt% BC is about 16 MPa, while PLA/BC bio-composites with 5 wt% biochar loading were brittle, and, thus, it was not technically possible to measure their mechanical properties. It appears that the tensile strength of PLA/BC composites, in both cases, is slightly increased in bio-composites with 1 wt% BC and decreased thereafter until 5 wt% biochar loading, at which point the specimens become brittle. The initial small increase could be attributed to the reinforcement effect of BC. A similar enhancement was also found in our previous study in poly(butylene succinate)/BC bio-composites [30], while the reduction thereafter could be attributed to the low molecular weight (Table 1). Incorporating biochar into a polymer matrix has been considered a method of improving its mechanical properties in the majority of the existing research that has been recently done [15,30,53]. More specifically, Mei Po et al. prepared PLA with bamboo charcoal composites with 2.5 wt%, 5 wt%, 7.5 wt%, and 10 wt% content. The increasing biochar content increases the mechanical properties, such as tensile strength and Young’s Modulus. Nevertheless, further addition of biochar content than 10 wt% results in decreasing the tensile strength [54]. Our results seem more compatible with the study of Mariem Zouari et al., wherein, adding biochar content leads to the reduction of tensile strength as well as young modulus, when biochar loading is more than 5 wt% [55].

Regarding the elongation at break, since PLA is a very brittle polymer with low elongation at break (about 1.3%), all bio-composites have similar values, as was expected. Furthermore, it was observed that the behavior of the elastic modulus of PLA/BC bio-composites has a similar trend as in the case of tensile strength. There is a negligible increase in PLA/BC bio-composites with 1 wt% BC and a significant reduction for PLA/BC when the concentration of BC is increased to 2.5 wt%.

### 3.9. Effect of Biochar Content on PLA Coloration

The increasing biochar content resulted in the coloration of PLA films. The color was measured with a colorimeter, and it is expressed with CIE coordinates in Table 4. PLA has an L* value of 88.98 and a* and b* values of −1.12 and 0.82, respectively, which is expected due to its white color. After the incorporation of both biochars, L* progressively decreased as expected due to their black color. The K/S fraction shows the concentration of the color on the composites, which increased with increasing biochar addition. PLA/SWP550 5% and PLA/SWP700 5% possess a higher concentration of the black color, 16.02 and 16.19, respectively. The color of composites changes due to the incorporation of biochar in a polymeric matrix. Similar findings were demonstrated by the study of Mariem Zouari et al., who investigated the addition of biochar to PLA and PLA/hemp fibers composites [18]. 

### 3.10. Enzymatic Hydrolysis

**Figure 11:**
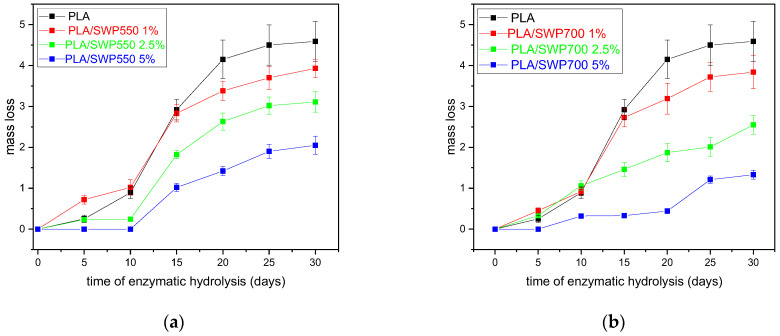
Mass loss vs. time plot during enzymatic hydrolysis of neat PLA and PLA/BC bio-composites, (**a**) with biochar at 550 °C, (**b**) with biochar at 700 °C.

As an aliphatic polyester, PLA occupies a relatively large number of ester bonds. For this reason, lipases, *Pseudomonas cepacian* (exotype) and *Rhizopus orizae* (endotype) can hydrolyze PLA and its bio-composites. The effect of enzymes on the mass loss of PLA and its bio-composites is presented in Figure 11. The mass loss of neat PLA is about 4% after 30 days of enzymatic hydrolysis. On the other hand, the incorporation of biochar into the PLA matrix leads to a decrease in mass loss rate. More specifically, as biochar content increases, the mass loss of bio-composites decreases. Bio-composites containing 1, 2.5, and 5 wt% SWP550 lost about 3.9, 3.1, and 2% of their mass, respectively, after 30 days of enzymatic hydrolysis, while bio-composites containing 1, 2.5, and 5 wt% SWP700 lost about 3.8, 2.5, and 1.3% of their mass, respectively.

The effect of the hydrophilicity or hydrophobicity of the composites was explored by the measurement of their water contact angle (Table 5). The water contact angle of polymers depends on their surface properties, the preparation method, and the roughness, as well as the chemical composition and the temperature [56]. PLA displayed a water contact angle value of around 73°, whereas the addition of biochar increased this value. As the biochar content increased to 5 wt%, the water contact angle reached 82°. The reduced hydrophilicity of the bio-composites can be primarily attributed to the biochar’s thermochemical conversion. Biochar loses the hydrophilic groups during the pyrolysis process, moving towards a more hydrophobic behavior. For this reason, its addition to PLA produces more hydrophobic bio-composites, which delay the enzymatic hydrolysis rate of PLA in all composites [18,55,57]. An increase in the content of both of the two types of biochar leads to a reduction in biodegradation time. Prolonged biodegradability of PLA can significantly increase the decomposition time and, thus, lead to the accumulation of this type of polymer in the environment, although it is a biobased and eco-friendly material. However, a longer biodegradability time may be useful for certain applications [58].

**Figure 12:**
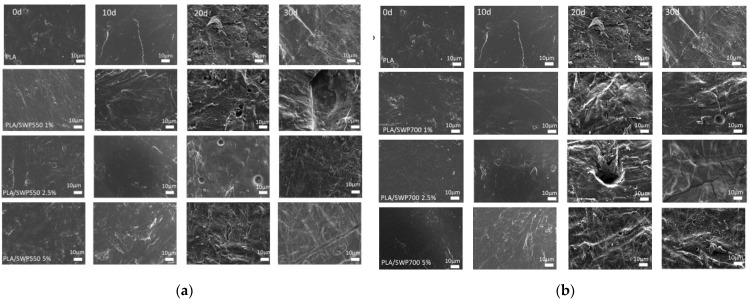
SEM micrographs of PLA and its bio-composites: (**a**) with biochar at 550 °C, (**b**) with biochar at 700 °C throughout enzymatic hydrolysis (10, 20, and 30 days).

The morphology of the sample’s surfaces after 30 days of enzymatic hydrolysis was evaluated using SEM micrographs (Figure 12). The surfaces of neat PLA and its bio-composites are smooth before the first days of enzymatic hydrolysis. The appearance of the surface’s deterioration becomes apparent after 20 days of hydrolysis in both cases of two different biochar pyrolysis types (SWP550 and SWP700) with the presence of roughness, ridges, and holes. These observations are in agreement with the mass loss measurements.

### 3.11. Thermal Degradation Studies of PLA/BC Bio-Composites

**Figure 13:**
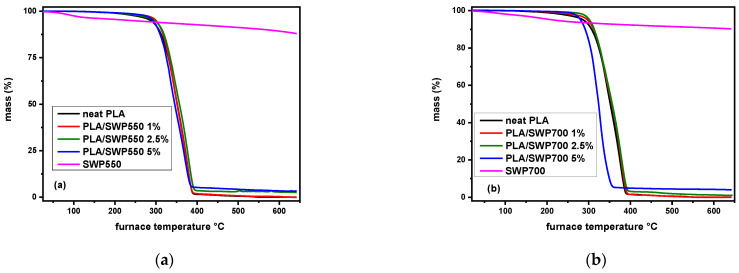

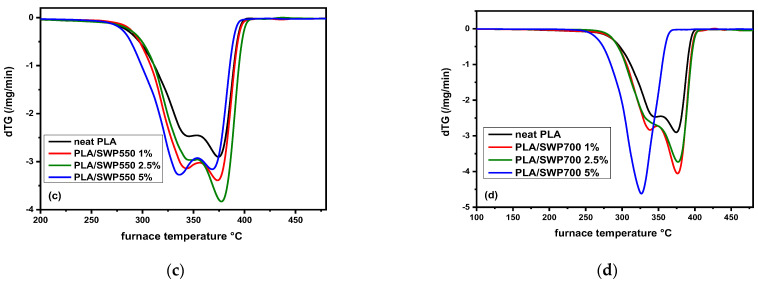
TGA thermograms and dTG curves of (**a**,**c**) PLA_SWP550 and (**b**,**d**) PLA_SWP700 at a heating rate of 20 °C/min under nitrogen flow.

The thermal degradation of PLA/BC bio-composites was tested by TGA. The thermal graphs and the derivative thermogravimetry (dTG) curves of the two PLA_SWP550 and PLA_SWP700 bio-composites, which were filled with varying filler content (1 to 5 wt% of BC), are shown in Figure 13, being heated with a heating rate of 20 °C/min under a nitrogen atmosphere. The SWP550 and SWP700 results represented that BC was the sample with the most stable thermal behavior, with only 12% of the mass loss at temperatures below 600 °C. This seems logically consistent since biochar is the result of preparing organic waste-designated fly ash via thermal treatment [59]. The TGA curves indicate that neat PLA and PLA BC bio-composites show good resistance to thermal degradation. The literature shows that variations in biochar stemming from different feedstocks can influence the thermal stability of the polymer matrix in different ways. Some researchers emphasize that biochar has a nucleating role in PLA [60]. Conversely, there are also findings that report biochar’s negative effect on thermal stability [15]. For example, Hernandez-Charpak et al. [16] found that the biochar produced from anaerobically digested dairy manure (DM) significantly decreased the crystallization and thermal stability of PLA, while biochar from eastern white pine wood chip (WC) had a much lesser impact on these properties. These variations have been associated with the moisture level of the DM and WC biochars, which facilitated the hydrolytic degradation of the polymer.

Table 6 shows the TGA results of neat PLA and PLA/BC composites with biochar of different contents, respectively. The temperatures corresponding to 1, 2.5, and 5 wt% mass loss and the maximum degradation temperatures (T_d,max1_ and T_d,max2_) indicate the effect of biochar and concentration on the thermal stability of PLA. The TGA results carry information that, in brief, neat PLA loses only 0.5% of the whole mass already at T_0.5_ and after that breaks majorly at 343.0 °C and 374.5 °C, which means that PLA composites made of SWP550 and SWP700 show the efficiency of even small biochar concentrations with T0.5 even reaching a peak, as indicated by T0.5. When 5 wt% SWP550 is added, the T_0.5_ slightly decreases to 161.1 °C, but the thermal stability improves at later stages with T_2.5_ and T_5_ shifting to 274.8 °C and 293.0 °C, respectively. The maximum degradation temperatures (T_d,max1_ and T_d,max2_) are somewhat lower, at 335.9 °C and 368.3 °C, when compared to neat PLA.

The initial thermal decomposition temperature of PLA/SWP700 5 wt% is 216.2 °C, and it indicates better early-stage thermal stability than the SWP550. However, in the case of T_5_ (280.8 °C) and maximum degradation temperature (326.5 °C), they are found to be significantly less than those for the PLA/SWP550 5 wt% composite, implying that the decomposition process might be further expedited due to the SWP700 loading effect. The data suggests that the biochar (SWP700) ingrains early-stage thermal resistance in PLA but catalyzes faster degradation at higher temperatures. This variance clearly shows how biochar exhibits diverse effects on PLA’s thermal properties, depending on its origin and the amount of biochar used. However, when the biochar content is increased, the composite is more resilient against thermal degradation. The residual mass also increased with more biochar added. This is because, at higher temperatures, more char residues make the composite stable. Similarly, in a previous study [61], it was also found that when the BC was added to the system, it made the layer denser, and therefore, more mass was left as residual mass. Additionally, the obtained data indicates an accelerating influence on the thermal stability of PLA/BC composites as filler content increases; thus, a catalytic effect of biochar in the degradation together with a faster degradation rate can be deduced from the above explanation. In the study of Arrigo et al. [15], a gradual decrease in T_5%_ and T_10%_ (the temperatures corresponding to 5% and 10% mass loss values) with increasing filler content was accompanied by a change in the maximum temperature at which degradation occurred. This decrease is explained by the presence of potassium, the main component in biochar, that speeds up the degradation process of PLA and other bio-polyesters. The report of Haeldermans et al. [60] indicated that the onset and maximum degradation temperatures of PLA/biochar composites are diminished with the increment of biochar content. With 20% biochar, these temperatures drop to around 290 °C and 365 °C, respectively, and further decline to 275 °C and 350 °C with 50% biochar. This is likely due to a reduction in molecular weight during processing, accelerated by the presence of fillers, as well as the catalytic effect of carbonates from the biochar on PLA’s ester bonds [18].

The degradation behavior of PLA biochar composites was analyzed by calculating the degree of conversion (α) and kinetic parameters using a combination of isoconversional methods and model-fitting approaches [35,61]. Following the kinetics of such a solid-state reaction may be defined as the mass left during the degradation process at the specific point that is assumed to be the result of the relationship between temperature and the conversion rate. Apart from that, another crucial parameter is the time in which the reaction model accounts for taking the reaction rate of arrival of gas species to fulfill the requirements, i.e., the rate of reaction constant (k) follows Arrhenius equation that describes the relation of the reaction speed with the temperature, the terms besides the Activation Energy (E in kJ/mol), gas constant (8.314 J/mol⋅K), and pre-exponential factor (A in s^−1^) that also contribute. Isoconversional methods are a set of techniques that are used to examine reaction kinetics, especially in the thermal degradation of materials. They assume that for a constant conversion level, the reaction rate is only temperature-dependent. They enable the determination of activation energy in the absence of a specific reaction model. The most popular isoconversional methods include differential methods like the Friedman method and integral methods such as Ozawa–Flynn–Wall (OFW), Kissinger–Akahira–Sunose (KAS), and Vyazovkin methods [62,63,64]. The Friedman method directly calculates activation energy without approximation, while the Vyazovkin method uses a more accurate integral approach, making it well-suited for complex reactions.

**Figure 14:**
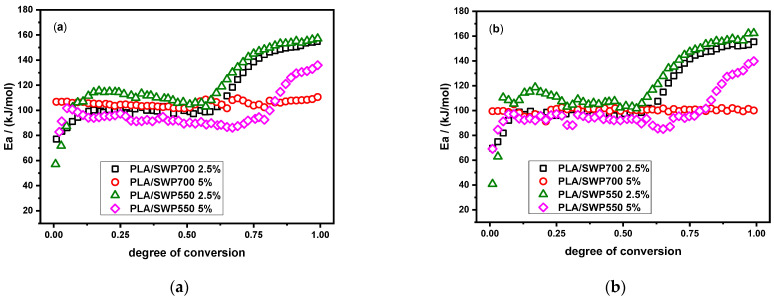
The dependence of activation energy (E_α_) on the degree of conversion (α) for the thermal degradation of PLA/SWP550 and PLA/SWP700 composites as calculated by (**a**) Friedman method and (**b**) Vyazovkin analysis.

Figure 14 illustrates the activation energy (E_α_) values with error bars for PLA/SWP550 and PLA/SWP700 composites as a function of the degree of conversion (α), utilizing the previously mentioned isoconversional methods. The average E_α_ values obtained using the Vyazovkin method from TGA data closely matched those calculated by the Friedman method. In all cases, the variation in E_α_ suggests that the thermal degradation mechanism is complex, with different processes influencing the kinetics at various stages of degradation.

The E_α_ for all samples, except PLA/SWP700 5 wt%, shows an initial rise, followed by relatively stable values in the intermediate range and then another increase in the final stage, indicating a complex degradation mechanism. Using both the Friedman and Vyazovkin methods, noticeable differences in E_α_ were observed across the different ranges of α. For PLA/SWP550 2.5 wt%, the mean E_α_ is approximately 75.1 kJ/mol (Friedman) and 78.3 kJ/mol (Vyazovkin) for α < 0.1. In the mid-range (0.1 < α < 0.6), the E_α_ increases to 109.9 kJ/mol (Friedman) and 108.1 kJ/mol (Vyazovkin), and for α > 0.6, it further rises to 141.8 kJ/mol and 141.3 kJ/mol. For PLA/SWP550 5 wt%, the E_α_ starts at 73.4 kJ/mol (Friedman) and 72.0 kJ/mol (Vyazovkin) for α < 0.1, rising to 93.8 kJ/mol (Friedman) and 94.1 kJ/mol (Vyazovkin) in the intermediate range, and increasing further to 129.7 kJ/mol (Friedman) and 128.5 kJ/mol for α > 0.6. For PLA/SWP700 2.5 wt%, the E_α_ values are around 83.4 kJ/mol (Friedman) and 78.1 kJ/mol (Vyazovkin) for α < 0.1, increasing to 100.4 kJ/mol (Friedman) and 98.8 kJ/mol (Vyazovkin) for the intermediate range, and reaching 140.7 kJ/mol (Friedman) and 139.2 kJ/mol for α > 0.6. In contrast, PLA/SWP700 5 wt% exhibits higher initial E_α_ values, averaging 105.9 kJ/mol (Friedman) and 100.9 kJ/mol (Vyazovkin) across all ranges of conversion.

PLA/SWP550 5 wt% shows a lower activation energy compared to PLA/SWP550 2.5 wt%, displaying a thermodynamic behavior that needs less E_α_ to start its thermal degradation response. The relatively lower activation energy indicates that the presence of SWP550 5 wt% particles impacts the degradation mechanism, which may promote higher susceptibility to thermal breakdown in a reinforced composite. PLA/SWP700 5 wt% shows a notably high activation energy, indicating strong thermal resistance, particularly in the early stages of degradation. The higher E_α_ suggests that more energy is required to initiate thermal breakdown, which implies that the composite is thermally stable in its initial phases. This stability is consistent with the TGA data in Table 1, where the onset degradation temperature (T0.5) for PLA/SWP700 5 wt% is 216.2 °C, significantly higher than other samples.

The E_α_ values for α ranges vary, indicating a mixed thermal degradation mechanism; however, it is highly interesting to note that the kinetics of degradation are determined by different mechanisms that are functional at different stages. A lot more importantly, the fact that E_α_ values went up at low and high levels while staying the same at intermediate levels and then going up again at the end shows that the degradation modes are very complexly connected during decomposition. In this sense, one of the principal contributions of isoconversional analysis is its influence on the selection of an appropriate multistep model.

An analytical model-fitting approach was applied in the study to determine the degradation mechanism and kinetic parameters, including the activation energy E_α_, the pre-exponential factor A, and the reaction model f(α), by using multivariate non-linear regression [61]. In addition, experimental data at four heating rates were compared with theoretical models and sixteen different reaction models were tested for compatibility with the experimental data. This was initially done in a one-step reaction model to account for the main mass loss, assuming average values as derived by isoconversional methods. Unless it fitted well, the methodology was extended by incorporating multiple reaction steps and refining the activation energy, drawing insight also from isoconversional methods, which gave much better results regarding the accuracy of the model.

**Figure 15:**
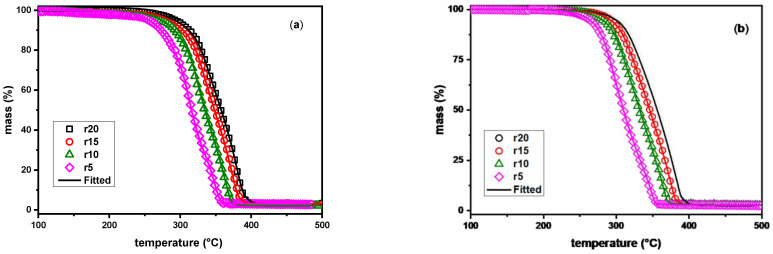
Mass (%) curves for selected (**a**) PLA/SWP550 2.5 wt% and (**b**) PLA/SWP700 2.5 wt% at heating rates of 5, 10, 15, and 20 °C/min in a nitrogen atmosphere (symbols) and corresponding fitted curves using the combination of Cn-Cn-Cn reaction models (Solid lines).

The three consecutive Cn-Cn-Cn reaction models better described the thermal degradation behavior of PLA/SWP550 2.5 wt%, PLA/SWP550 5 wt%, and PLA/SWP700 2.5 wt%. Figure 15 shows results for selected samples PLA/SWP550 2.5 wt% and PLA/SWP700 2.5 wt%.

The autocatalytic mechanism n-order (Cn) is mathematically expressed as:

(3) $$f\left(\alpha \right)={\left(1-\alpha \right)}^{n}(1+K_{cat}X)$$

where K_cat_ is the autocatalysis rate constant and X is the extent of conversion of the autocatalytic reactions. The three-step model effectively captures the degradation process, providing a more accurate representation of the conversion function compared to a single- or dual-step mechanism. The Cn-Cn-Cn model suggests that the degradation involves multiple stages where autocatalysis plays a key role, with each stage governed by its respective autocatalytic reaction. Using the consecutive Cn models reflects the gradual step-by-step break of the polymer chains in the presence of the biochar. In contrast, the PLA/SWP700 5 wt% sample is well described by a single Cn model, indicating that its degradation is less complex and can be effectively modeled by a simpler reaction mechanism. The PLA/SWP700 5 wt% sample presents one degradation step likely due to the increased carbon content and changes in biochar properties as a result of the higher pyrolysis temperature (700 °C). With the increase in pyrolysis temperature from 550 °C to 700 °C, the biochar undergoes significant structural transformations. During pyrolysis (especially at higher temperatures), biochar undergoes carbonization, which increases its carbon content. This process also reduces the presence of oxygen-containing functional groups like carboxylic acids, which are known to contribute to thermal instability due to their tendency to facilitate degradation reactions [65]. This single-step degradation suggests that the biochar produced at 700 °C creates a more stable interface with PLA, limiting multi-step breakdowns that are typically seen in composites with biochar produced at lower pyrolysis temperatures (like 550 °C). The differences in the number of Cn reactions required for each sample highlight the impact of biochar type and content on the degradation kinetics, with higher biochar content generally leading to more straightforward degradation behavior.

The experimentally determined parameters received in the tested models are listed in Table 7. On the one hand, composites in the PLA/BC data set showed high values of R^2^, which means they are strongly correlated with the experiment results and, therefore, are a very good fit for the model. The E_α_ values inferred from the multi-step mechanism approach compare favorably with those obtained by isoconversional methods (Figure 2). The thermal degradation process of the PLA biochar composites, specifically PLA/SWP700 2.5 wt% and PLA/SWP550 5 wt%, can be conceived as a complex multi-step mechanism due to the variation in activation energy at different stages of degradation. According to the activation energy in the first step (89.8 kJ/mol), PLAB/SWP700 2.5 wt% degradation is associated with simpler processes, and it can be theorized to be the breakdown of weaker molecular bonds or the less stable components of the composite that are initiating the degradation. The second stage, with a higher activation energy of 109.5 kJ/mol and a greater contribution of 0.617, is the stage of more energy-intensive reactions, perhaps by the transformation of more stable polymer regions or the development of stronger interactions between PLA and biochar. The last stage, which has an activation energy of 137.9 kJ/mol, is the sign of the critical period when the most thermally stable parts of the composite are breaking down. In the case of PLA/SWP550 2.5 wt%, a similar sequence is found with a slightly lower activation energy in the first stage, which reflects the cruciality of biochar’s low pyrolysis temperature. The first step (87.2 kJ/mol) is the one depicting the easiest initiation of degradation, whereas the second phase (108.3 kJ/mol) denotes the existence of considerable thermal resistance in the intermediate stages. The last step, with an activation energy of 140.4 kJ/mol, reveals a much higher breakdown of substances that demands the use of the most energy. The rising activation energy through the stages of degradation, along with higher Log Kcat values, highlights the role of autocatalysis in accelerating the reaction, especially in later stages. The autocatalytic effect is more pronounced in the biochar-biopolymer composites, where the biochar particles are the catalyst that catalyzes the breakdown of the PLA chains, particularly at higher temperatures. It is the relation between PLA and biochar as well as the properties of biochar itself (such as surface area, porosity, and the existence of functional groups) that alter the degradation way.

The introduction of 5 wt% biochar to the PLA composites has a much more evident change in the thermal degradation behavior, which is evident through low Eα values and a corresponding increase in the decomposition rate constant. This accelerated decomposition might be referred to as the higher the biochar content, which acts as a catalyst for the easier thermal breakdown; due to the more BC particles, the quicker degradation starts. The A is also lower for PLA/BC 5 wt% composites in comparison to PLA/BC 2.5 wt%. This decrease in A is due to the reduced Eα and the fact that fewer energies are needed for the degradation reactions in the higher biochar content composite. Additionally, the larger rate constant for PLA/BC 5 wt% suggests that the degradation process is happening more rapidly. This comes from the fact that the extra biochar has led to a huge promoter in the surface area and thus more catalytic activity, permitting faster polymer chain scission and hence the acceleration of the overall thermal degradation process. The explanation for this is that the reduction in the activation energy additionally means that the energy barriers for degradation are removed, making the composite breakdown easier by thermal stress.

In comparison, PLA/BC 2.5 wt%, with higher Eα and pre-exponential factor values, demonstrates greater thermal stability. The higher energy barrier for degradation in this composite suggests that fewer polymer chains are breaking down at lower temperatures, resulting in slower overall thermal decomposition. This highlights the inverse relationship between biochar content and thermal stability, where increasing BC content enhances degradation but reduces the composite’s resistance to thermal breakdown.

This behavior emphasizes the dual role of biochar in PLA composites: while it reinforces the material at lower concentrations, providing stability and improved thermal properties, higher concentrations act as catalysts, promoting quicker degradation by lowering the activation energy and increasing the rate constantly.

## 4. Conclusions

PLA bio-composites containing two different types of biochar, SWP550 and SWP700, which have been produced by pyrolysis of softwood pellets at 550 and 700 °C, were prepared by in situ ROP. From the TEM micrographs, it was found that this technique is appropriate for bio-composite preparation since it produces a sufficiently good dispersion of BC in the polymeric matrix. GPC measurements verified that PLA with high molecular weight can be synthesized, which has been progressively reduced in the bio-composites as biochar content increased. This reduction was attributed to the strong interactions between BC and PLA or due to the ability of BC’s hydroxyl groups to act as initiators for ring-opening polymerization of lactide. The alteration in the ester group vibrations that are recorded in FTIR spectra indicates the interactions between BC and PLA polymeric matrix. These interactions led to a systematic deceleration of segmental mobility of bio-composites in combination with the elevation of the T_g_. Furthermore, the aforementioned results are responsible for the slight suppression of the PLA bio-composites crystallizability. Bio-composites with SWP700 show a slightly stronger effect compared to SWP550. The different load of biochar and in different pyrolysis temperatures enhances slightly tensile strength at low BC content (1 wt%) while at higher loading, mechanical properties deteriorated. This could be attributed to the lower molar mass of the bio-composites. The loading of biochar, in both cases, reduced the hydrolysis rates of PLA bio-composites due to the slightly higher hydrophobicity of these samples. From TGA analysis, it was found that different types of biochar exhibit diverse effects on PLA bio-composite thermal properties.

## Figures and Tables

**Table 1:** Intrinsic viscosity and molecular weight of the prepared PLA and its bio-composites.

Sample	η (dL/g)	M_n_ (g/mol)	PDI
PLA neat	2.18	109,000	2.2
PLA/SWP550 1 wt%	1.26	40,200	1.53
PLA/SWP550 2.5 wt%	0.92	30,150	1.66
PLA/SWP550 5 wt%	0.8	22,500	1.71
PLA/SWP700 1 wt%	1.14	40,900	1.60
PLA/SWP700 2.5 wt%	0.99	30,800	1.81
PLA/SWP700 5 wt%	0.72	20,150	1.60

**Table 2:** The samples under investigation, values of interest: molar mass, M_n_, glass transition temperature, T_g_, change in heat capacity, Δc_p_, melt/hot crystallization temperature and crystalline fraction, T_c_ and CF_c_, cold crystallization temperature and crystalline fraction, T_cc_ and CF_cc_, melting temperature(s), T_m_. Note: CF_i_ was estimated by comparing the normalized enthalpy change (ΔH_i,norm_) with the heat of fusion for PLA, taken as ΔH_100%_ = 93 J/g.

		Scan 1 (Fast Cooling ~100 K/min)					Scan 2 (Cooling at 20 K/min)						
Sample	*M*_n_ (kg/mo)	*CF*_c_ (wt)	*T*_g_ (°C)	Δ*c*_p_ (J/g∙K)	*T*_cc_ (°C)	*CF*_cc_ (wt)	*T*_c_ (°C)	*CF*_c_ (wt)	*T*_g_ (°C)	Δ*c*_p_ (J/g∙K)	*T*_cc_ (°C)	*CF*_cc_ (wt)	*T*_m_ (°C)
neat PLA	109	0	46	0.50	89	0.55	126/91	0.35	44	0.26	87	0.09	167
PLA/SWP550 1.0%	40	0	52	0.49	103	0.47	111/97	0.08	52	0.45	103	0.38	175
PLA/SWP550 2.5%	30	0	47	0.52	93	0.54	92	0.25	45	0.28	89	0.24	167
PLA/SWP550 5.0%	23	0	53	0.51	98	0.53	94	0.19	54	0.32	95	0.26	156/172
PLA/SWP700 1.0%	41	0	47	0.50	94	0.47	92	0.12	49	0.39	95	0.30	170
PLA/SWP700 2.5%	31	0	56	0.47	106	0.51	93	0.03	56	0.47	104	0.44	157
PLA/SWP700 5.0%	20	0	54	0.53	124	0.46	-	0	55	0.51	124	0.46	161/164

**Table 3:** Mechanical properties of PLA and its bio-composites.

Sample	Tensile Strength at Break (MPa)	Elongation at Break (%)	Elastic Modulus (MPa)
PLA	30.08 ± 1.09	1.28 ± 0.07	3037 ± 231
PLA/SWP550 1 wt%	32.01 ± 0.34	1.51 ± 0.09	3143 ± 104
PLA/SWP550 2.5 wt%	15.25 ± 0.27	1.32 ± 0.02	2300 ± 111
PLA/SWP700 1 wt%	31.08 ± 0.72	1.12 ± 0.05	3187 ± 176
PLA/SWP700 2.5 wt%	16.82 ± 0.81	1.68 ± 0.03	1883 ± 59

For the samples PLA/SWP550 5 wt% and PLA/SWP700 5 wt% there are no available data.

**Table 4:** Colorimetric data L*, a*, b*, C*, h°, and K/S values of PLA and PLA/BC samples.

Samples	L	a	b	C	h	K/S
PLA	88.98	−1.12	0.82	1.39	143.77	0.13
PLA/SWP550 1 wt%	30.40	0.44	1.34	1.16	64.17	9.02
PLA/SWP550 2.5 wt%	23.50	0.31	0.62	0.39	49.17	11.93
PLA/SWP550 5 wt%	19.81	0.15	0.13	0.22	321.62	16.02
PLA/SWP700 1 wt%	21.33	0.30	0	0.18	41.11	13.04
PLA/SWP700 2.5 wt%	20.34	0.36	−0.13	0.16	312.63	15.46
PLA/SWP700 5 wt%	19.91	0.12	0.04	0.14	347.69	16.19

**Table 5:** Contact angle measurements of PLA and its bio-composites.

Sample	Contact Angle (Degree)
PLA	73.90 ± 0.40
PLA/SWP550 1 wt%	73.80 ± 0.39
PLA/SWP550 2.5 wt%	76.70 ± 0.44
PLA/SWP550 5 wt%	82.50 ± 0.42
PLA/SWP700 1 wt%	72.70 ± 0.42
PLA/SWP700 2.5 wt%	75.20 ± 0.36
PLA/SWP700 5 wt%	80.00 ± 0.39

**Table 6:** TGA results of all samples studied.

Sample	*T*_0.5_ (°C)	*T*_2.5_ (°C)	*T*_5_ (°C)	*T*_d,max1_ (°C)	T_d,max2_ (°C)
Neat PLA	162.3	250.0	286.8	343.0	374.5
PLA/SWP550 1 wt%	172.4	276.9	297.2	343.2	373.5
PLA/SWP550 2.5 wt%	166.4	262.7	296.5	343.8	377.2
PLA/SWP550 5 wt%	161.1	274.8	293.0	335.9	368.3
PLA/SWP700 1 wt%	181.5	272.8	297.0	337.2	376.1
PLA/SWP700 2.5 wt%	210.8	290.8	302.0	332.4	376.7
PLA/SWP700 5 wt%	216.2	270.2	280.8	-	326.5

**Table 7:** Activation energy, pre-exponential factor, and reaction order of all studied samples.

Sample	Model	Activation Energy, E/kJmol^−1^	Pre-Exponential Factor, logA_1_/s^−1^	Reaction Order/n	Log K_cat_	Contribution	R^2^
PLA/SWP700 2.5%	Cn	89.8	7.5	0.3	0.7	0.242	0.99996
	Cn	109.5	10.0	0.8	0.8	0.617	
	Cn	137.9	11.2	1.6	1.3	0.141	
PLA/SWP700 5%	Cn	104.9	6.9	1.2	0.9	1.000	0.99990
PLA/SWP550 2.5%	Cn	87.2	6.4	0.4	0.3	0.266	0.99973
	Cn	108.3	9.8	0.7	0.6	0.574	
	Cn	140.4	11.0	1.7	1.0	0.160	
PLA/SWP550 5%	Cn	79.0	5.3	0.3	0.7	0.251	0.99954
	Cn	103.6	9.2	0.6	0.9	0.501	
	Cn	133.64	10.2	1.3	1.1	0.248	

## Data Availability

All the data of this study are included in the manuscript.
